# IL-33-primed human mast cells drive IL-9 production by CD4^+^ effector T cells in an OX40L-dependent manner

**DOI:** 10.3389/fimmu.2024.1470546

**Published:** 2024-10-02

**Authors:** Louise Battut, Edouard Leveque, Salvatore Valitutti, Nicolas Cenac, Gilles Dietrich, Eric Espinosa

**Affiliations:** ^1^ Université Toulouse III – Paul Sabatier, FSI, Toulouse, France; ^2^ Inserm, U1220, Institut de Recherche en Santé Digestive (IRSD), INRAE, INP-ENVT, Toulouse, France; ^3^ Centre de Recherche en Cancérologie de Toulouse (CRCT), INSERM UMR1037, CNRS UMR5071, Toulouse, France; ^4^ Department of Pathology, Institut Universitaire du Cancer-Oncopole de Toulouse, CHU Toulouse, Toulouse, France

**Keywords:** mast cell, IL-33, OX40L, Th cell, IL-9

## Abstract

Interleukin-33 (IL-33) is an alarmin released by epithelial cells in response to tissue damage. It activates resident immune sentinel cells, which then produce signals commonly associated with type 2 immune responses, particularly affecting infiltrating antigen-specific T cells. Given that mast cells (MCs) are a primary target of IL-33 and can shape T helper (Th) cell responses, we investigated the effect of IL-33 priming on the ability of MCs to influence Th cell cytokine production. To examine the Th cell/MC interaction, we developed human primary MC/memory CD4^+^ T-cell coculture systems involving both cognate and non-cognate interactions. Our results demonstrated that IL-33-primed MCs, whether as bystander cells cocultured with activated effector T cells or functioning as antigen-presenting cells, promoted IL-9 and increased IL-13 production in Th cells via an OX40L-dependent mechanism. This indicates that MCs sense IL-33-associated danger, prompting them to direct Th cells to produce the key type 2 effector cytokines IL-9 and IL-13.

## 1 Introduction

Type 2 immune responses are set up in response to major tissue damage in the skin and mucosa, typically against macroparasites or environmental insults (1, 2). Noxious stimuli are sensed in the epithelium and induce the release of alarmins such as thymic stromal lymphopoietin (TSLP), interleukin-25 (IL-25), and IL-33 alarmins that prime tissue-resident sentinel cells and induce the maturation of dendritic cells that next mount Th2 cell response in the draining lymph node (3–5). Once infiltrated in the injured tissues, Th2 cells receive confirmation signals provided by the immunological milieu that not only reinforce but also mold their polarization and lead to a subtle combination of type 2 cytokine production (IL-4, IL-5, IL-9, and IL-13) within the damaged tissue (2, 3, 6). These cytokines regulate several defense and repair modules promoting resistance and tolerance mechanisms (7). Local professional or occasional APCs play a key role in the restimulation of Th cells. It was reported that DC priming by IL-33 during antigen presentation promotes IL-5, IL-13, and IL-9 production in Th cells (8, 9). Moreover, group 2 innate lymphoid cells (ILC2s) were shown to foster local Th2 response via direct interactions with Th2 cells (10–12). Besides ILC2 and dendritic cells, mast cells (MCs) are also known to promote Th2 responses, but the underlying mechanism remains poorly understood.

MCs are sentinel innate immune cells particularly abundant in skin and mucosa that, like ILC2s, are primed by alarmins and notably by IL-33. IL-33 belongs to the IL-1 family cytokines and binds a heterodimeric receptor consisting of ST2 (IL-1RL1) and its coreceptor IL-1 receptor accessory protein (IL-1RAcP). Signal transduction pathways are initiated by the recruitment of Myd88 and IL-1R-associated kinase (IRAK) family members to activate several downstream signaling pathways such as MAPKinases and NFkB (13–15). MCs highly express ST2 (16, 17) and are strongly stimulated by IL-33 promoting cell maturation, and cytokine and chemokine production (18–22). MCs are known to influence adaptive immune responses and appear as tunable cells leading to activatory or inhibitory effects on T-cell responses according to the immunological circumstances (23, 24). Because we and others previously showed that MCs can play the role of occasional APCs and shape CD4^+^ Th cell response (25–27), we investigated in this study the effects of IL-33 on human MC abilities to interact with Th cells and influence their polarization. We showed that IL-33-primed MCs fostered Th2 cell responses and allowed IL-9-producing Th cells to emerge in an OX40L-dependent manner.

## 2 Methods

### 2.1 Human primary MC cultures

Human peripheral blood mononuclear cells (PBMCs) were isolated by density centrifugation (Ficoll-Paque PLUS, Cytiva) from buffy coats of healthy human donors aged between 18 and 66. Buffy coats were provided anonymously by the Etablissement Français du Sang, Toulouse, France. CD34^+^ precursor cells were isolated from the PBMCs by positive selection with anti-CD34 magnetic beads (EasySep™ Human CD34 Positive Selection Kit, STEMCELL Technologies). CD34^+^ cells were grown in serum-free StemSpan™ medium (STEMCELL Technologies) supplemented with recombinant human IL-6 (50 ng/mL; Peprotech), recombinant human IL-3 (10 ng/mL; Peprotech), and 3% supernatant of CHO transfectants secreting murine SCF (a gift from Dr. P. Dubreuil, Marseille, France; 3% corresponds to 50 ng/mL SCF) for 1 week at 37°C, 5% CO_2_. Cells were next grown in IMDM GlutaMAX-I supplemented with sodium pyruvate, 2-mercaptoethanol, 0.5% BSA, insulin-transferrin selenium, penicillin/streptomycin (100 U/mL/100 µg/mL) (all from Invitrogen), IL-6 (50 ng/mL), and 3% supernatant of CHO transfectants secreting murine SCF for 8 weeks and tested phenotypically (CD117^+^, FcϵRI^+^, ST2^+^) and functionally (β-hexosaminidase release in response to FcεRI crosslinking) before being used for experiments. Only primary cell lines exhibiting more than 95% CD117^+^/FcϵRI^+^/ST2^+^ cells were used for experiments.

### 2.2 Coculture experiments

A total of 1 × 10^5^ memory CD4^+^ T cells freshly purified from PBMCs by immunomagnetic negative selection (EasySep™ Human memory CD4^+^ T cell enrichment kit, STEMCELL Technologies) were cocultured at a 1:1 ratio with human primary MCs (primed or not with 10 ng/mL of human recombinant IL-33 for 4 h and next washed) for 6 days in the presence of magnetic beads coated with anti-CD3 and anti-CD28 (anti-CD3/CD28 beads) mAbs (Dynabeads, Life Technologies) at 1 bead:10 T cells ratio in coculture medium [RPMI 1640 supplemented with 10% serum replacement medium (knockout medium, Life Technologies), GlutaMAX-I, sodium pyruvate, 2-mercaptoethanol, and 1% supernatant of CHO transfectants secreting murine SCF].

To induce an APC phenotype, primary human MCs were treated with recombinant human INF-γ (50 ng/mL, Peprotech) for 48 h at 37°C. MCs were next washed and primed with recombinant human IL-33 (10 ng/mL, Peprotech) for 4 h at 37°C. Subsequently, MCs were washed again and either pulsed or not with a cocktail of superantigens (50 ng/mL TSST-1, SEA, SEB, SEE, Sec-1; Toxin Technology, Sarasota, Florida) for 2 h. After two additional washes, 1 × 10^5^ MCs were cocultured at a 1:1 ratio with memory CD4^+^ T cells for 6 days in RPMI 1640 medium supplemented with 10% serum replacement (knockout medium, Life Technologies), GlutaMAX-I, sodium pyruvate, 2-mercaptoethanol, and 1% supernatant from CHO transfectants secreting murine SCF. When indicated, cocultures were treated with anti-OX40L mAb (10 µg/mL, R4930 Oxelumab, Novus Biologicals) or Ig control (10 µg/mL, Normal Rabbit IgG Control, R&D Systems). At day 6, cells were processed for intracellular cytokine staining.

### 2.3 Flow cytometry

#### 2.3.1 Cell surface molecule staining

Cells were incubated with fluorochrome-labeled primary antibodies [anti-CD117-PECyanin7 (clone 104D2, BioLegend), anti-CD54-BV786 (clone HA58, BD Biosciences), anti-HLA DR/DP/DQ-BUV395 (clone Tu39, BD Biosciences), and anti-OX40L-PE (clone ik-1, BD Biosciences)] in PBS, 1% FCS, and 1% human serum (HS) (FACS buffer) according to the manufacturer’s recommended concentration, at 4°C for 30 min. Cell viability was assessed by using fixable viability dye eFluor 780 (eBioscience).

#### 2.3.2 Intracellular cytokine staining

To determine the cytokine profile of the coculture resulting T cells, cells were re-stimulated with PMA (50 ng/mL) and ionomycin (1 µg/mL) in the presence of GolgiPlug™ and GolgiStop™ (BD Biosciences), or GolgiStop only for IL-9 measurement, for 5 h. After washing in PBS, cells were incubated with anti-CD117-PECyanin7 (clone 104D2, BioLegend) and viability dye in FACS buffer at 4°C for 30 min. Cells were washed and fixed in 4% PFA for 15 min at room temperature (RT) and permeabilized with PBS, 1% FCS, 1% HS, and 0.1% saponin (permeabilization buffer) for 10 min. Cells were next incubated with the indicated Abs: anti-IL-17A-BV510 (clone BL168), anti-IFN-γ-FITC (clone B27), anti-IL-4-APC (clone 8D4-8), anti-IL-13-BV711 (clone JES10-52A), and anti-IL-9-PE (clone MH9A4) in permeabilization buffer for 45 min at RT (all from BD Biosciences). After PBS wash, counting beads (CountBright™, Life technologies) were added to measure cell numbers during flow cytometry acquisition. All flow cytometry experiments were acquired using a Fortessa flow cytometer and the FACS Diva software (BD Biosciences) and analyzed with FlowJo V10 software (TreeStar).

### 2.4 Cytokine quantitation

To evaluate CD4^+^ T-cell cytokine production (IFN-γ/IL-17/IL-4/IL-13/IL-9) post-coculture, CD4^+^ T cells were sorted by FACS (FACSAria or FACSMelody cell sorter, BD Biosciences) (CD117^-^, SSC^low^) at day 6 and restimulated with PMA (50 ng/mL) and ionomycin (1 µg/mL) for 5 h. Supernatants were harvested and stored at −80°C. Cytokine concentrations were measured using a bead-based multiplex assay (LEGENDplex™, BioLegend) following the manufacturer’s recommendations. Flow cytometric acquisitions were performed with a MACSQuant^®^ Analyzer 10 instrument (Miltenyi Biotec) and analyzed with FlowJo V10 software (TreeStar).

### 2.5 Immunofluorescence and confocal microscopy

For MC staining, cells were plated on poly-L-lysine (Sigma)-coated slides and fixed in 4% PFA for 15 min at RT and next blocked/permeabilized with PBS, 1% BSA, and 0.1% saponin (blocking buffer) for 30 min at RT. They were incubated with mouse anti-tryptase (IgG1 kappa, clone AA1, Dako) and anti-OX40L (polyclonal rabbit IgG, Invitrogen) at RT for 1 h in blocking buffer. MCs were washed and incubated with matched donkey AlexaFluor-conjugated secondary antibodies (Invitrogen) at RT for 1 h in blocking buffer and next counterstained with DAPI (1 µg/mL, Invitrogen).

For immunological synapse (IS) staining, SAg-pulsed or unpulsed MCs and CD3/CD28 pre-activated (24 h) CD4^+^ memory T cells were mixed at a 1:1 ratio and centrifuged at 70*g* for 30 s in a U-bottom 96-well plate and incubated for 10 min at 37°C in coculture medium to allow conjugate formation. They were next gently pipetted and plated on poly-L-lysine-coated slides for 7 min before fixation in methanol for 15 min at −20°C. Cells were blocked/permeabilized in blocking buffer for 30 min at RT and subsequently stained with mouse anti-OX40 mAb (IgG1 kappa, clone ACT35, BD Pharmingen), rabbit anti-OX40L (polyclonal rabbit, PA5-116057, Invitrogen), and anti-CD4 (polyclonal goat, AF-379-NA, R&D Systems). They were next incubated with matched AlexaFluor-conjugated secondary antibodies (Invitrogen) and counterstained with DAPI. Images were acquired using a Zeiss LSM 780 confocal microscope and analyzed using Zen (Carl Zeiss Microscopy) and ImageJ software.

### 2.6 Intercellular communication score computation

To infer the helped MC-activated CD4^+^ memory T-cell communication network, we based our analysis on our previous study where MCs were stimulated by IL-33 for 4 h and processed for RNAseq analysis (GEO accession number GSE235240) using the publicly available database of ligand–receptor pairs collated by Noel and co-workers (28) and a computation method previously described (28, 29). A thresholding method was used to infer “active” L–R pairs, and communication score computation (expression product method) was used to rank the active L–R pairs. Briefly, average expression levels (FPKM) of the genes corresponding to the ligands in the database were selected from the list of DEGs between IL-33-primed and resting MCs (FDR <0.01 and log_2_ fold change >1). Average expression levels (mean FPKM value) of the genes corresponding to the receptors in the database were selected from RNAseq data of memory CD4+ T cells activated with anti-CD3/CD28-coated beads for 24 h (GEO accession number GSE73214) (30). For thresholding, only ligands and receptors with FPKM > 1 were kept in the analysis. Intercellular communication score was calculated as previously described (28): ligand and receptor gene expression were next scaled by the maximum value of gene expression among ligands and receptors, respectively, and multiplied by 10 to obtain values ranging from 0 to 10. The maximum value of gene expression was calculated as the mean of the 10% highest values. Scaled values above 10 (outliers) were coerced to 10. Because the database employed here takes into account multichain receptors or ligands, we first calculated the expression level for the multichain molecules as the geometric average of the values of the ligand/receptor chain expression. Next, the ligand–receptor pair score was determined by the product of the expression level of the ligand by the expression level of the receptor (if L_i_ is the average expression level of ligand i by helped MC and R_i_ is the average expression level of the corresponding receptor by activated CD4^+^ memory T cell, the score S_i_ of this interaction is S_i_ = L_i_.R_i_). Inferred active ligand–receptor pairs (S_i_ > 0) are provided in **Supplementary Table S1**. Custom R scripts were used for analyses. For circular visualization of the links between MC^IL-33^ ligands and activated memory CD4^+^ T-cell corresponding receptors, the circlize R package was used (31) to represent the 30 top-ranked L–R pairs according to their computed communication score.

### 2.7 Statistical analysis

Statistical analyses for all experiments were conducted in GraphPad Prism 9 (GraphPad Software) or with rstatix and PMCMRplus R packages. Data were first analyzed for normality with Shapiro–Wilk test and variance homogeneity with Levene’s test. To determine statistical significance in assays comparing more than two groups, data showing homogeneity of variance and normality were analyzed with repeated-measures one-way ANOVA followed up by paired pairwise *t*-test with Bonferroni correction, otherwise with Friedman test followed up by pairwise Wilcoxon signed-rank tests with Bonferroni correction. All *p*-values are two-sided (*p < 0.05; **p < 0.01; ***p < 0.001; ****p < 0.0001; ns, not significant).

## 3 Results

### 3.1 IL-33-primed MCs drive IL-9 production in activated CD4^+^ Th cells

**Figure 1:**
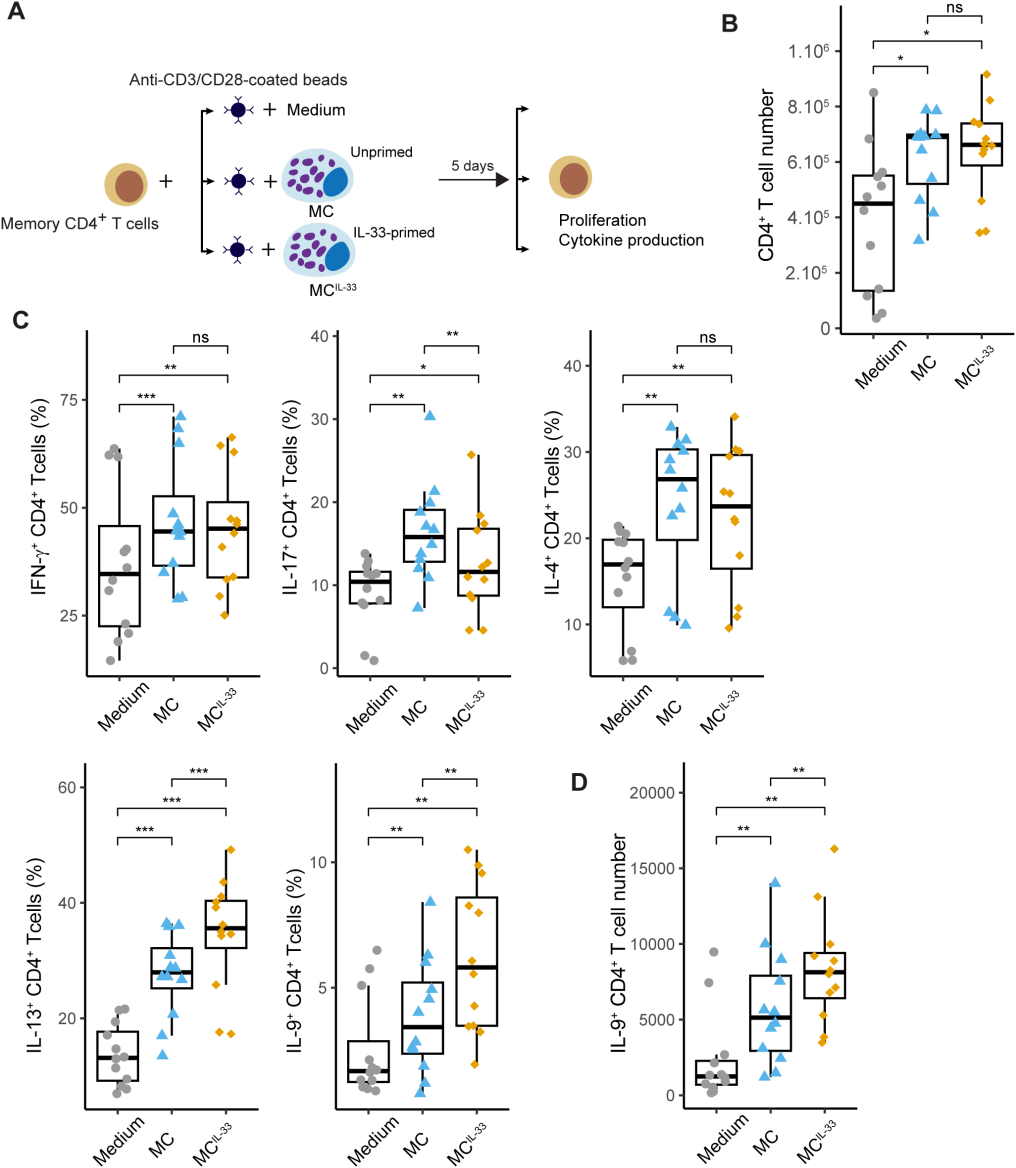
IL-33-primed MCs drive IL-9 production in activated CD4^+^ Th cells. **(A)** Experimental settings: freshly isolated memory CD4^+^ T cells were stimulated with anti-CD3/CD28-coated beads in the presence or absence of MCs primed or not with IL-33 for 6 days. Cells were next analyzed by flow cytometry for intracellular cytokine staining. **(B)** CD4^+^ T cell numbers measured at day 6 of coculture. **(C)** Percentage of CD4^+^ staining positive for indicated cytokines. **(D)** IL-9^+^CD4^+^ T-cell numbers measured at day 6 of coculture. Points represent *n* = 12 independent experiments. Box-and-whiskers plots in the style of Tukey. *p < 0.05, **p < 0.01, ***p < 0.001; ns, not significant.

To analyze the effects of IL-33 priming on MCs and their subsequent influence on Th cell responses, we devised a coculture system in which freshly isolated peripheral blood CD4^+^ memory T cells were stimulated with anti-CD3/CD28 beads to mimic antigenic recall in the presence of allogeneic human MCs (pure primary culture derived from peripheral blood precursors) either primed or unprimed with IL-33 (**Figure 1A**). Coculture with MCs, regardless of IL-33 priming, increased the proliferation (**Figure 1B**) and early (CD69 and CD25) or late (CD137) activation marker expression on the CD4^+^ T-cell surface (**Supplementary Figure S1**), suggesting that MCs provide activating signals to CD4^+^ T cells independently of IL-33. We next analyzed typical Th cell cytokine (IFN-γ, IL-17, IL-4, IL-13, and IL-9) production by CD4^+^ T cells after 6 days of coculture. We observed that unprimed MCs increased the percentages of CD4^+^ T cells producing these cytokines, indicating that MC presence facilitates T-cell cytokine production (**Figure 1C**). MCs primed with IL-33 (MC^IL-33^) significantly increased the percentages of IL-9^+^ (and, to a lesser extent, IL-13^+^) CD4^+^ T cells and decreased IL-17^+^ T-cell percentage without affecting the proportions of IFN-γ^+^ or IL-4^+^ T cells (**Figure 1C**). Moreover, the number of IL-9^+^ cells increased in the presence of MC^IL-33^(**Figure 1D**). Together, these results demonstrate that IL-33 enhances the capacity of MCs to drive Th cells toward IL-13 and IL-9 production.

### 3.2 OX40L expression in IL-33-primed MCs promotes IL-9 production in Th cells

Because IL-33 endows MCs with the capability to foster IL-9 production by Th cells, we sought to elucidate the underlying mechanism of this phenomenon. In a previous study, we analyzed the transcriptome of primary human MCs stimulated with IL-33 using RNA sequencing (GSE235240) (32), revealing that IL-33 alters the expression of more than 2,600 genes. Here, we investigated whether the genes upregulated in MCs following IL-33 stimulation might play a role in the interaction between MCs and activated memory CD4^+^ T cells.

**Figure 2:**
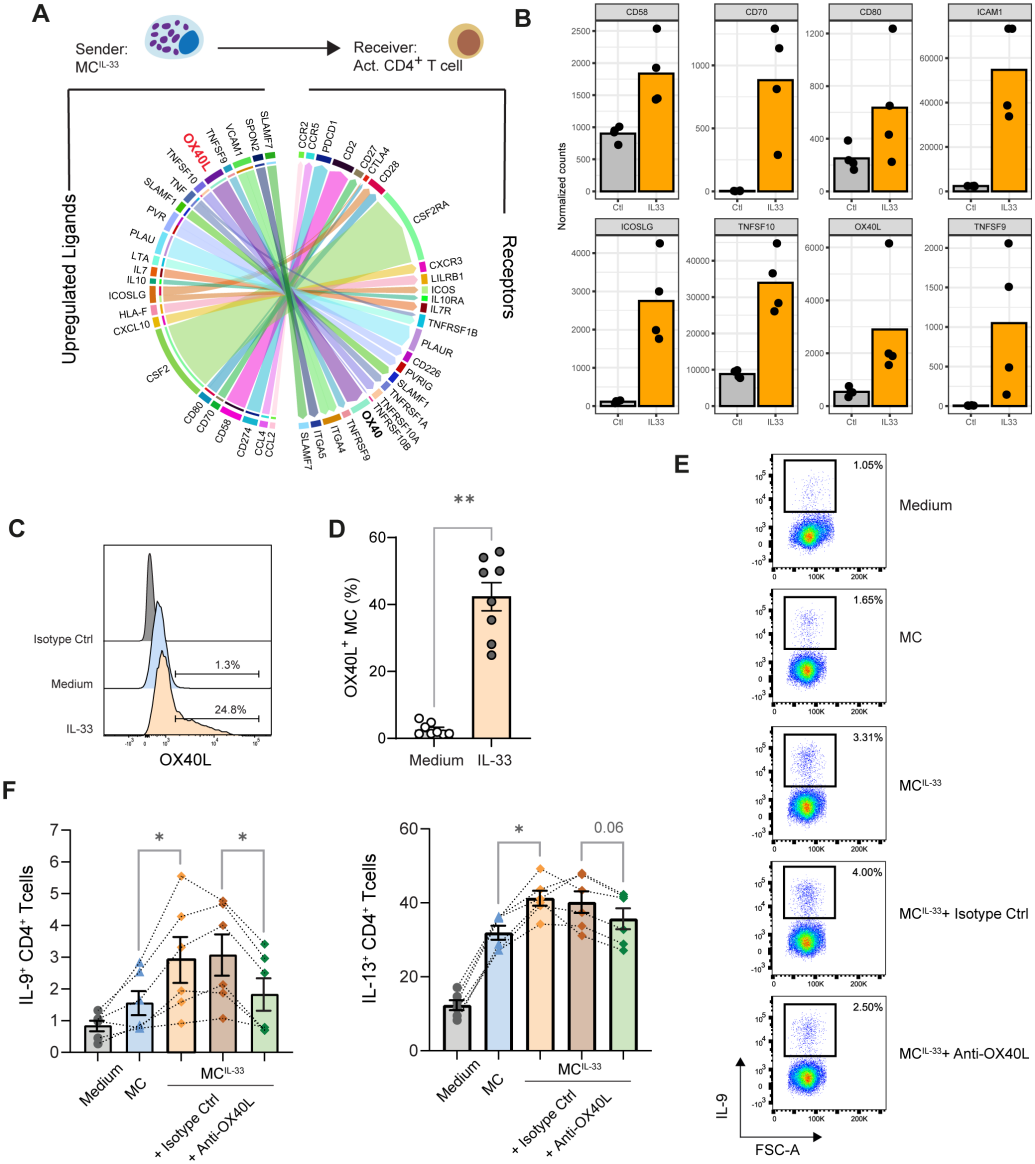
OX40L induced by IL-33 in MCs promotes IL-9 production in Th cells. **(A)** Ligand–receptor interaction inference and visualization. The top 30 strongest predicted interactions visualized by the circle plot depicting links between predicted ligands upregulated in MCs upon IL-33 treatment with their associated receptors expressed in memory CD4^+^ T cells activated with anti-CD3/CD28 beads. The arrow thickness is proportional to the computed L–R score. Multiple ICAM-1/integrin interactions have the highest score but are not represented to avoid overloading the diagram. Some gene symbols have been replaced by more commonly used protein names. **(B)** Expression of indicated costimulation molecules and TNFSF molecules by MC treated or not with IL-33 according to the RNAseq dataset. **(C, D)** OX40L expression on the MC surface following IL-33 treatment analyzed by flow cytometry; representative histogram **(C)** and pooled data from *n* = 8 donors are shown; bars represent mean ± SEM; each point corresponds to an experiment, Wilcoxon matched-pairs signed rank test. **(E, F)** Analysis of cytokine production by memory CD4^+^ T cell stimulated with anti-CD3/CD28-coated beads in the presence of MCs or MC^IL-33^ or MC^IL-33^ plus anti-OX40L blocking mAb or its isotype matched control. Representative dot plots of indicated intracellular cytokine stainings and pooled data from six independent experiments are shown. Bars represent mean ± SEM, and each point corresponds to an independent experiment, Friedman test followed up by pairwise Wilcoxon signed-rank tests. *p < 0.05, **p < 0.01.

To this end, we analyzed putative MC-activated memory CD4^+^ T-cell communication based on MC ligand expression upregulated by IL-33 in our RNAseq dataset and on receptor expression in memory CD4^+^ T cells activated for 24 h with anti-CD3/28 beads retrieved in the publicly available dataset (30). We computed a communication score for each L–R pair in a publicly available database by adapting the computational method described in the ICELLNET framework (28) dedicated to infer cell–cell communication. This score is based on both the expression level thresholding method (to determine active L–R pairs) and the R–L expression product method (to rank the active L–R pairs) (29) with MCs as sender cells and activated Th cells as receiver cells. This analysis inferred 53 active L–R pairs (**Supplementary Table S1**), suggesting that IL-33 upregulated in MCs the expression of several molecules involved in T-cell costimulation such as CD80, CD58, ICAM1, and TNF superfamily (TNFSF) molecules CD70 (TNFSF7), OX40L (TNFSF4), and CD137L (TNFSF9) (**Figures 2A, B**). Among these molecules, we focused on OX40L because it was shown to be associated with Th2 promotion (10, 33, 34).

We confirmed by flow cytometry that IL-33 induced OX40L expression on the MC surface (**Figures 2C, D**). Because other type 2 response alarmins might prime MCs and it was reported that in human CD11c^+^ DCs express OX40L in response to TSLP but not to IL-33 (33), we analyzed the impact of MC priming by TSLP and IL-25 and observed that these alarmins did not induce OX40L expression on the MC surface (**Supplementary Figure S2**). To test the effect of OX40/OX40L costimulation on Th cell polarization, we used anti-OX40L blocking mAb oxelumab. The addition of oxelumab significantly impaired MC’s ability to increase the percentage of IL-9^+^ Th cells (**Figures 2E, F**). A trend to reduce IL-13^+^ Th cell proportion in the presence of oxelumab was also noted. This result indicates that OX40 stimulation is required to promote IL-9 (and to a lesser extent IL-13) production by Th cells in our coculture system.

### 3.3 IL-33-primed antigen-presenting MCs drive IL-9 production in Th cells

Because we and others previously showed that IFN-γ induced an APC phenotype in MCs (MHC class II and costimulatory molecule expression) (27, 35), we tested whether IL-33 also acts on MCs’ capability to polarize Th cells upon cognate interactions. We induced an APC phenotype in MCs by stimulating them with IFN-γ for 48 h and analyzed the effect of IL-33 priming on their phenotype (CD54, HLA-class II molecules and OX40L expression). IL-33 synergized with IFN-γ to induce MHC class II molecules and CD54 but IFN-γ did not affect OX40L expression on the MC surface (**Supplementary Figure S3**).

**Figure 3:**
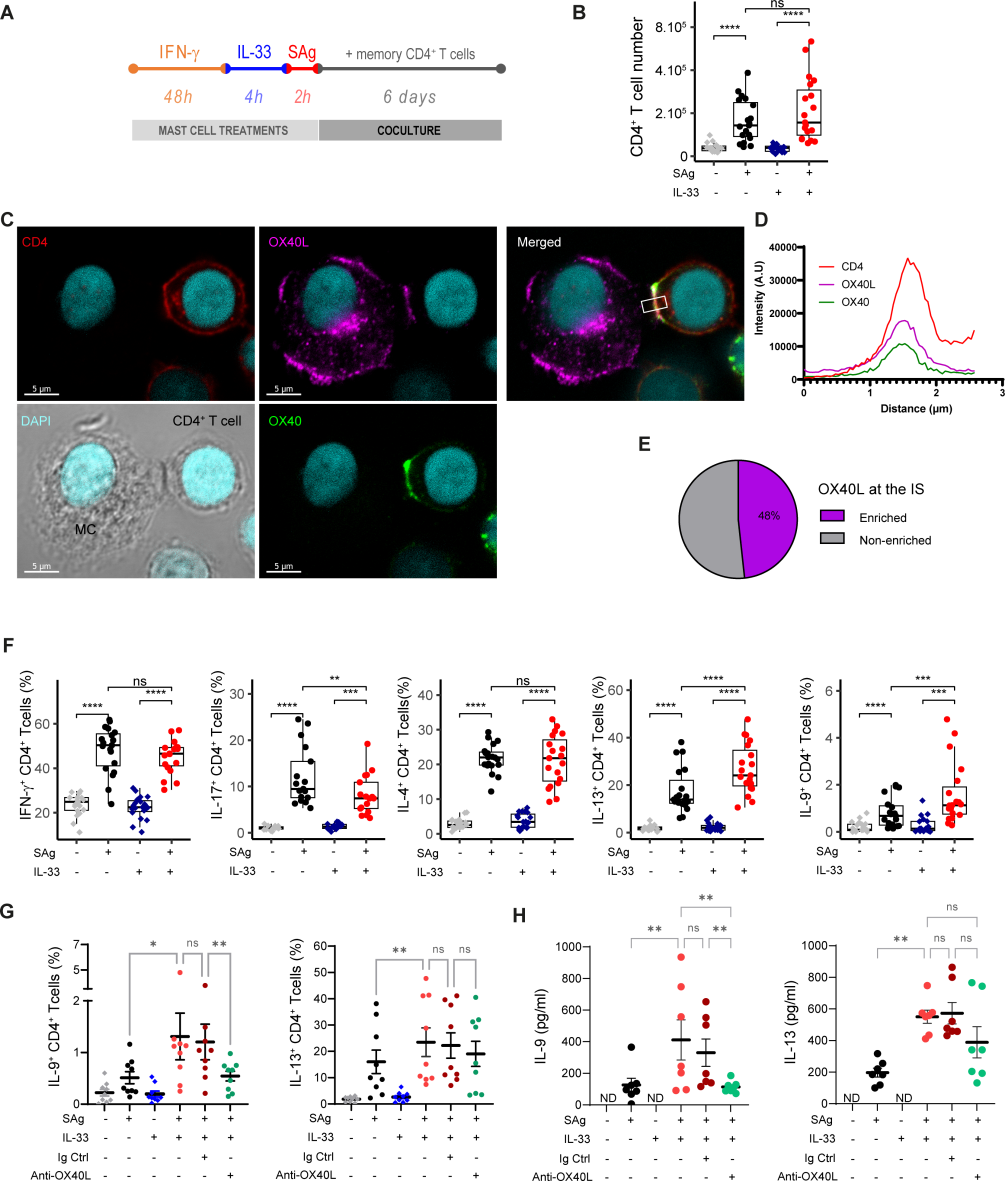
Antigen-presenting MCs primed with IL-33 promote IL-9 production in CD4^+^ T cells in an OX40L-dependent manner. **(A)** Experimental setting of coculture experiments. MCs were stimulated with IFN-γ for 48 h, treated with IL-33 for 4 h, and then loaded with a cocktail of SAg. MCs were cocultured for 6 days with freshly isolated human effector/memory CD4 T cells. **(B)** CD4^+^ T-cell number at the end of the coculture **(C–E)** Immunological synapse (IS) formation between SAg-pulsed MC^IL-33^ and memory CD4+ T cells, typical example of OX40L enrichment at the IS **(C)**, fluorescence intensity profile of channels corresponding to CD4, OX40, and OX40L staining averaged along the rectangle drawn in **(C, D)**, quantification of MC/CD4^+^ T-cell conjugates showing OX40L enrichment or not at the IS (*n* ~ 50) **(E)**. **(F, G)** Intracellular flow cytometry analysis of CD4^+^ T-cell cytokine production **(F)**, in the presence of anti-OX40L blocking mAb or rabbit IgG control **(G)**. **(H)** Amount of IL-9 and IL-13 produced by FACS-sorted CD4^+^ T cells after 6 days of coculture and restimulated by PMA/ionomycin. Box-and-whiskers plots in the style of Tukey or mean ± SEM; each point corresponds to an independent experiment. *p < 0.05, **p < 0.01, ***p < 0.001, ****p<0.0001; ns, not significant.

IFN-γ-primed MCs were pulsed with a cocktail of bacterial superantigens to activate a substantial fraction of the polyclonal memory CD4^+^ T-cell population (approximately 70%) (36) and cocultured with CD4^+^ memory T cells for 6 days (**Figure 3A**). We observed that SAg-pulsed MC efficiently stimulated polyclonal T-cell proliferation and that IL-33 priming did not change MCs’ capacity to stimulate CD4^+^ T-cell proliferation (**Figure 3B**), indicating that IL-33 priming did not change the potential of MCs to activate Teff cells. To check whether MC^IL-33^–Th cell cognate interactions did occur upon coculture and whether OX40L was involved at the IS, we analyzed IS formation by confocal laser scanning microscopy 2 h after coculture. No conjugates were observed with IFN-γ-primed MC^IL-33^ unpulsed with SAg. Approximately 10% of IFN-γ-primed MC^IL-33^ pulsed with SAg were found conjugated with memory CD4^+^ T cells after 2 h of coculture, indicating that MC^IL-33^–CD4^+^ T-cell cognate interactions did occur. Conjugates were analyzed for OX40L and OX40 enrichment at the cell–cell contact area. We observed that approximately half of the conjugates showed OX40L enrichment at the IS, suggesting the involvement of Th cell OX40 costimulation upon cognate interaction with MCs (**Figures 3C–E**). We next analyzed cytokine production by Th cells at day 6 of coculture (**Figure 3F**). IL-33-primed MCs showed an enhanced capacity to increase the proportion of IL-9^+^ and IL-13^+^ Th cells and to reduce the percentage of IL-17^+^ Th cells (**Figure 3F**). The addition of blocking anti-OX40L mAb indicated that IL-9^+^ Th cell differentiation required OX40–OX40L interaction (**Figures 3G, H**). Taken together, these results show that MC^IL-33^ pretreated with IFN-γ are capable of stimulating memory CD4^+^ T cells in a cognate manner and to foster IL-9^+^ Th cells via OX40L expression.

## 4 Discussion

In this study, we showed that IL-33 endows MCs with the ability to drive memory CD4^+^ T cells toward IL-9 and IL-13 production in an OX40L-dependent manner. Our results indicated that this phenomenon occurs whether MC^IL-33^ act as APCs or as bystander cells for memory CD4^+^ T cells.

We observed that IL-33 induced several costimulation molecules or synergized with IFN-γ to equip MCs with antigen presentation-associated molecules such as MHC class II molecules and ICAM-1. Four-hour treatment with IL-33 led to a robust expression of OX40L on the human MC surface. OX40L was previously shown to be expressed in mouse bone marrow-derived MCs (37) or to be induced by FcεRI crosslinking in human MCs (38). OX40L binding to its receptor OX40 induces TNF receptor associated factor (TRAF) signaling pathways that synergize with TCR or cytokine-derived stimulation to foster NFκB pathway and sustain survival, proliferation, and cytokine production in T cells (34, 39, 40). It was proposed that MCs can express OX40L, allowing them to interact with CD4^+^ T lymphocytes, promoting their activation (37, 38). Our study put forward an IL-33/MC/OX40L axis in the fostering of type 2 response and more precisely of IL-9 production by Th cells.

OX40L is mainly expressed by professional APCs but was also found to be expressed by several cell types (34). It was reported that OX40L can be induced by TSLP in dendritic cells (41, 42) or by IL-25 in eosinophils (43). We showed that among type 2 alarmins TSLP, IL-25, and IL-33, only IL-33 is able to induce OX40L expression in MCs. This preferential induction of OX40L by IL-33 alarmin we observed in MCs was also reported in ILC2s by Halim et al. (12). Nevertheless, irrespective of its induction manner, OX40L expression by professional or non-professional APCs was associated with Th2 responses (8, 33, 44, 45). Likewise, our study includes MCs in this group of APCs that respond to type 2 alarmins by acquiring the ability to promote type 2 immune response.

Our findings suggest that costimulatory signal OX40L can be provided by MC^IL-33^ to Th cells independently of MCs’ capacity to present Ag. This could expand the scope of MC ability to promote IL-13 and IL-9 production in Th cells beyond their IFN-γ-dependent APC role. It indicates that, through OX40L expression triggering, IL-33 can involve MCs in type 2 Th cell response promotion even if conditions for MC to express MHC-II molecules and present antigen are not met. Our results suggest that MCs might play a similar role reported for ILC2s. In an experimental model of CD4^+^ T cells/ILC2 co-culture, ILC2s were shown to promote Th2 differentiation in the absence of antigenic stimulation but in an OX40L-dependent manner (10). In addition, it has been shown that ILC2s can serve as APCs and that ILC2s pulsed with OVA peptide polarized OT-II CD4^+^ T cells towards a Th2 phenotype (11, 46). Like ILC2s, MCs are tissue-resident non-professional APCs that might locally reactivate Ag-experienced Th cells, allowing and even molding their cytokine production (26, 27). We showed here that this ability can be tuned by the priming received by MCs, a mechanism well known in dendritic cells. IL-33 priming turns MCs into pro-type 2 cells and notably into IL-9 production promoters in memory Th cells as it was previously reported for dendritic cells (9). In agreement with Xiao et al.’s work, OX40L expression in MC^IL-33^ is the main driver of IL-9 expression in Th cells (45). Furthermore, it would be intriguing to investigate whether basophils are also capable of promoting IL-9 production by T helper cells, given their response to IL-33 (47) and ability to influence T helper cell polarization (48).

We did not observe an increase in the percentage of IL-4^+^ Th cells when MCs were primed with IL-33. This result echoes a previous report showing that dendritic cells primed with IL-33 induced IL-5 or IL-13 but not IL-4 in naive CD4^+^ T cells (8, 49). Because the presence of unprimed MCs dramatically increased the proportion of IL-4-producing Th cells, we can hypothesize that pro-IL-4 signaling in memory Th cells is already at its maximum level with unprimed MCs and cannot be increased. On the other hand, OX40 signaling would not raise IL-4 expression in memory T cells.

Literature survey shows that the IL-33/OX40L/Th2 axis might be different in mice and humans. Notably, C57BL/6 mice MCs do not express OX40L in response to IL-33 ((37) and our own observations). These discrepancies might underestimate the involvement of MC in type 2 immune responses in studies using the C57BL/6 strain. Furthermore, laboratory mice have a significantly lower number of MCs in their lung parenchyma compared to wild mice (50), which could result in an underestimation of the early MC role in priming immune responses in the lungs in the context of allergy. This could explain why ILC2s were the sole cells reported to express OX40L in the C57BL6 mouse model (12). The investigation of this IL-33/MC/OX40L axis in humans is challenging, yet some indications suggest that it may be operational. For instance, OX40L^+^ MCs were observed in contact with OX40^+^ T cells in the skin of patients with atopic dermatitis (51) or were reported in the context of alopecia areata (52) and gastric cancer (53). Moreover, interrogating sc-RNAseq analysis results obtained by Vieira Braga et al. and publicly available in the human lung atlas database https://www.lungcellatlas.org/ indicates that OX40L expression is increased in lung MCs from patients with asthma (**Supplementary Figure S4**) (54).

Our results integrate MCs in the immune response elicited by IL-33. In addition to enhancing MC functions and pro-inflammatory capabilities, IL-33 equips MCs with costimulation molecules, among which OX40L fosters IL-13 and IL-9 production by Th2 cells. Likewise, IL-33-licensed MCs could provide cues required by infiltrated Th cells to produce their cytokines and also signals that mold the panel of produced cytokines, here IL-9 and IL-13, via OX40–OX40L interaction.

## Data Availability

The original contributions presented in the study are included in the article/**Supplementary Material**. Further inquiries can be directed to the corresponding author.
