# Supplementary material for: IL-33-primed human mast cells drive IL-9 production by CD4+ effector T cells in an OX40L-dependent manner
Source: Front Immunol. 2024 Oct 2;15:1470546. doi: 10.3389/fimmu.2024.1470546 (PMC11479898; doi:10.3389/fimmu.2024.1470546)
Supplement: Supplementary file 1 [file DataSheet1.docx]

**Supplementary Data**


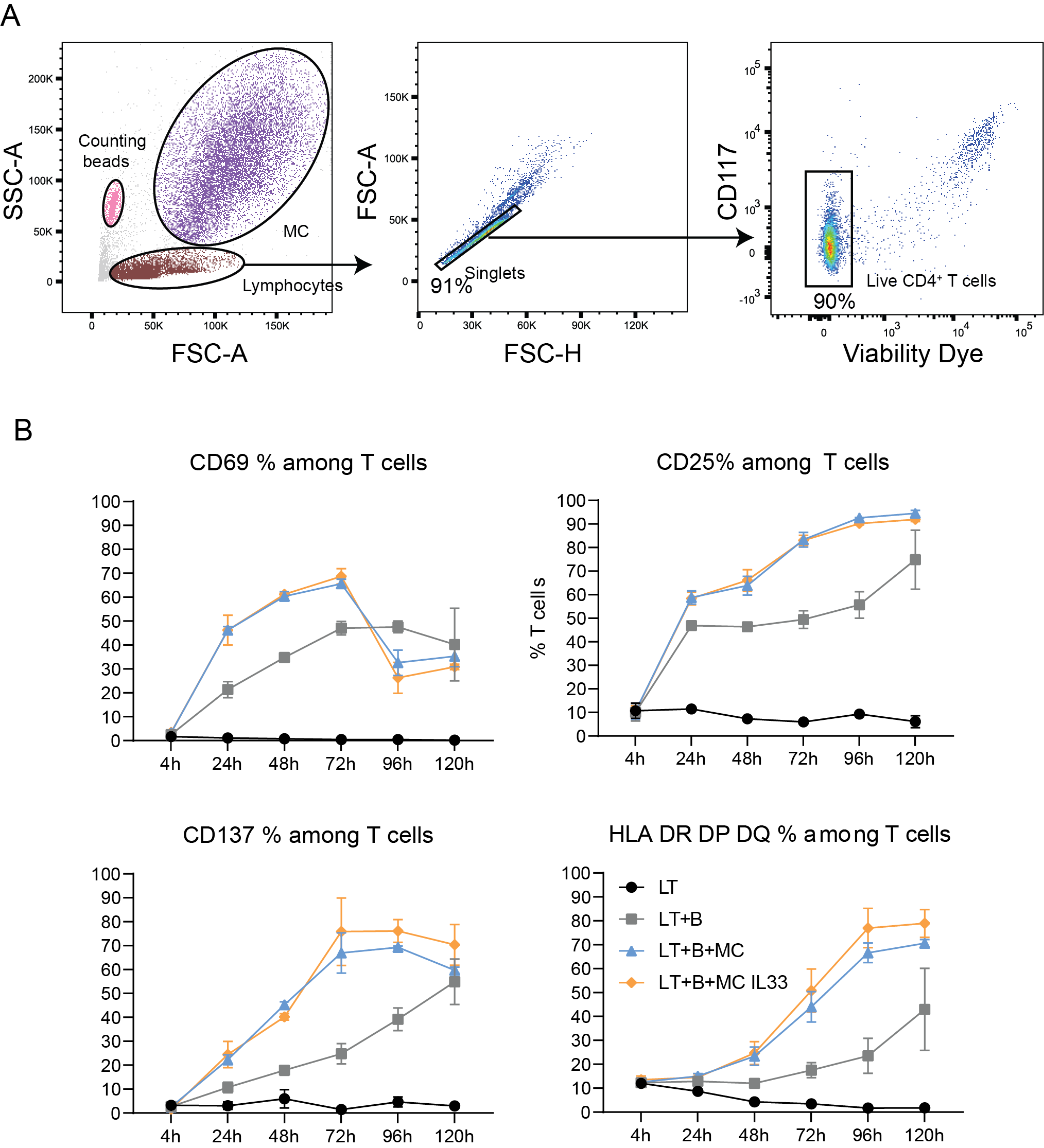


**Figure S1. Early and late activation marker expression by CD4^+^ T cells upon coculture with MCs.** CD4^+^ memory T cells were stimulated or not with anti-CD3/CD28 beads and cocultured or not with MCs or MC^IL33^. Cells were harvested at indicated time points and processed for immunofluorescence and flow cytometry analysis. **(A)** gating strategy, **(B)** flow cytometry analysis of CD69, CD25, HLA-DR/DP/DQ and CD137 on CD4^+^ T cell surface. points represent mean+/- SEM from 3 independent experiments.


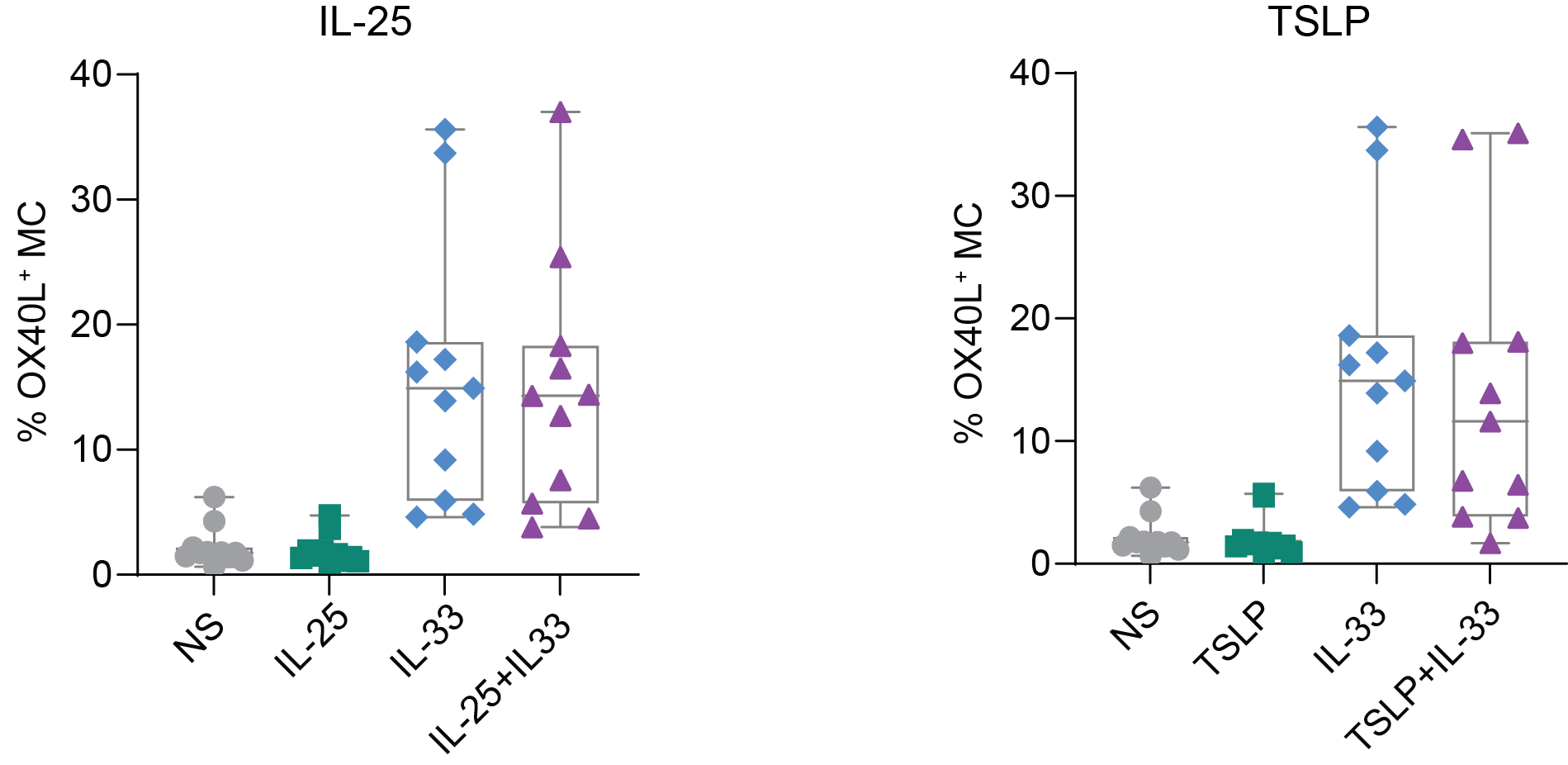


**Figure S2. Neither IL-25 nor TSLP induces OX40L expression at the MC surface.** MCs were stimulated with 10 ng/mL of IL-33, TSLP or IL-25 (alone or in combination) for 16 hours and OX40L expression was analyzed by flow cytometry. Each point represents an independent experiment (n=11).


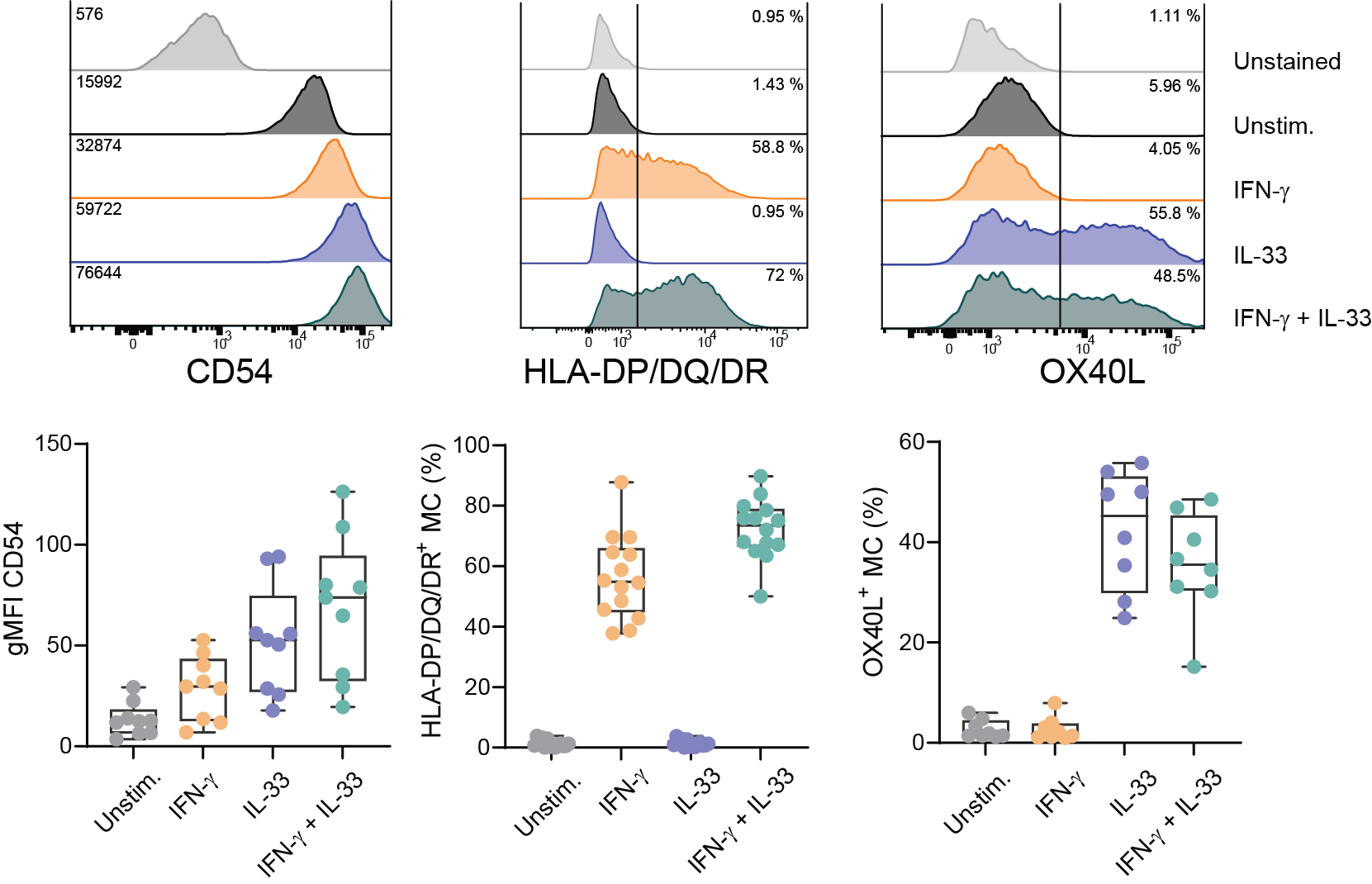


**Figure S3. Effects of IL-33 priming on CD54, MHC-class II molecules and OX40L expression on MC surface.** Primary human MCs were stimulated for 48 hours with IFN-γ and next treated with IL-33 for 4 hours. MHC class II molecules (HLA-DP/DQ/DR), CD54 (ICAM-I) and OX40L expressions were determined by flow cytometry. Shown are representative histograms and pooled data from n=8-12 donors.


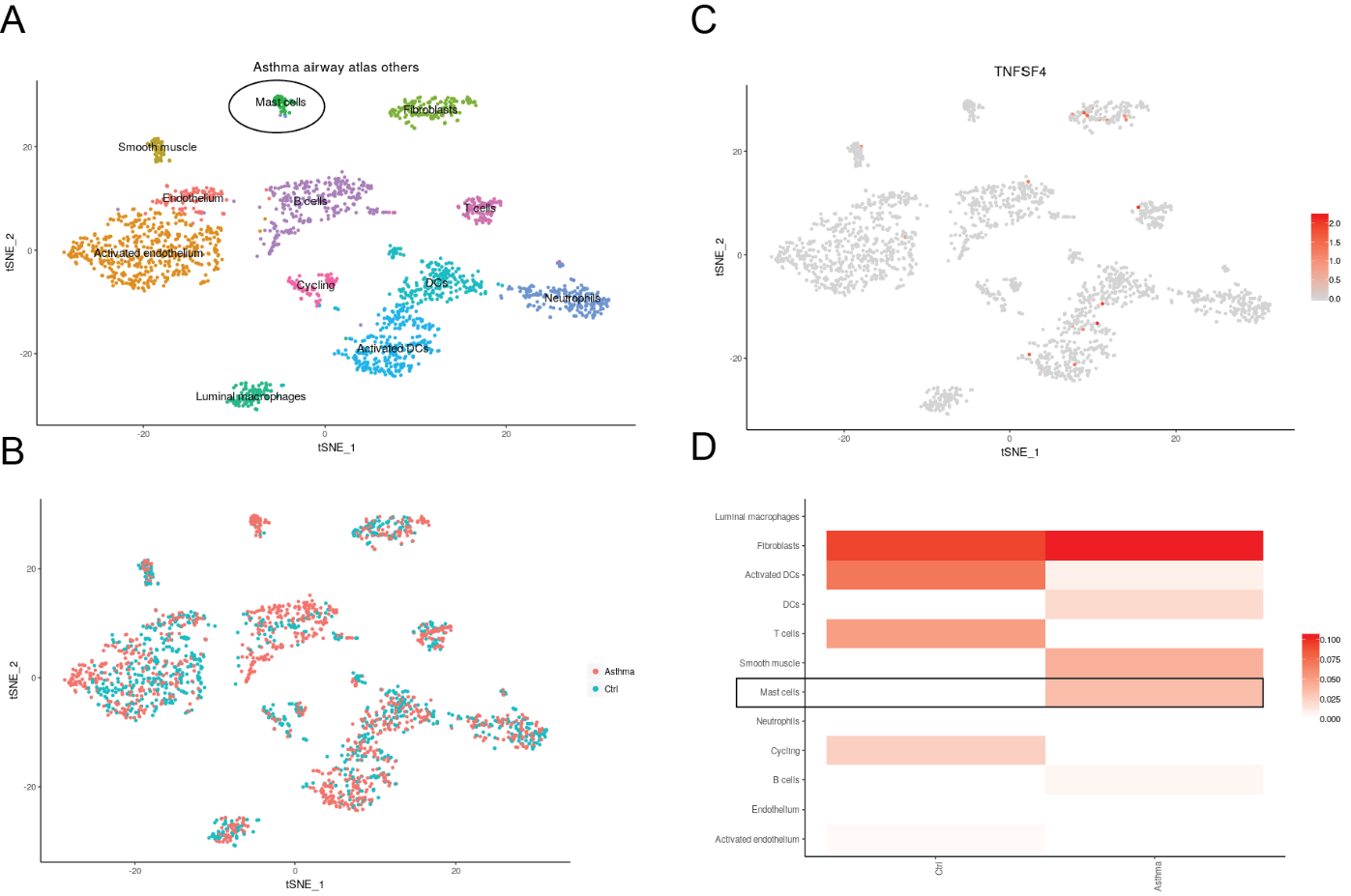


**Figure S4. TNFSF4 (OX40L) expression in single-cell RNAseq dataset from Vieira Braga et al. (in lungcellatlas.org).** scRNAseq 10x and smartseq2 from lungcellatlas.org. Airways and parenchyma, healthy and asthmatic patients. **(A)** cell groups identification, **(B)** Identification of cell origin between Ctrl and asthma patients, **(C)** TNFSF4 expression, **(D)** TNFSF4 expression level in cells from Ctrl and asthma patients.


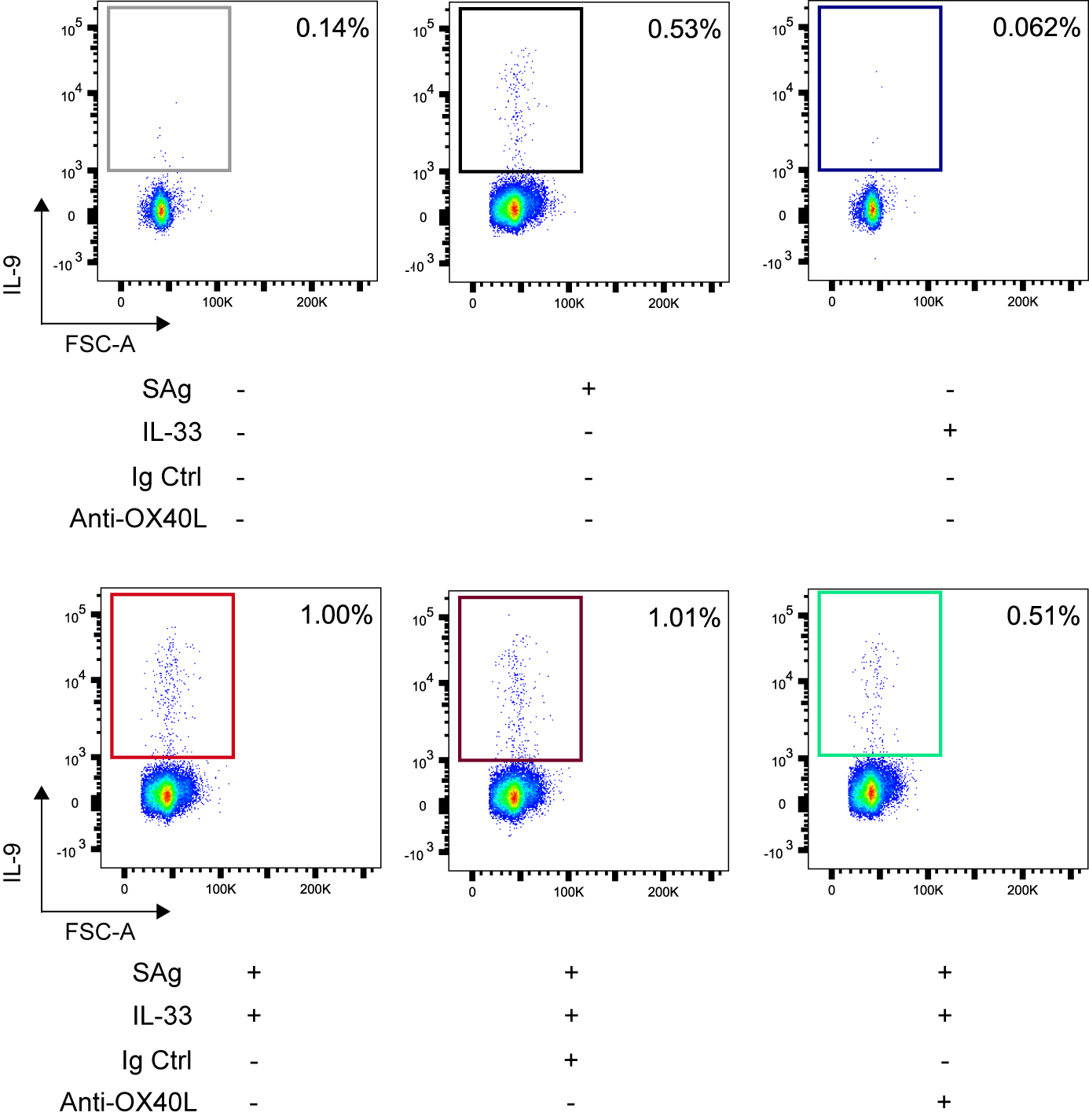


**Figure S5. Representative dotplot of IL-9 production by memory CD4^+^ T cell stimulated** **in the indicated conditions.** MCs were stimulated with IFN-γ for 48 hours, treated with IL-33 for 4 hours and next loaded with a cocktail of SAg. MCs were cocultured for 6 days with freshly isolated human memory CD4^+^ T cells and, when indicated, treated with anti-OX40L blocking mAb or rabbit IgG control.

**Table S1 Ligand-Receptor interaction inference**

predicted ligands from MC^IL-33^ (only ligands corresponding to DEGs from MC+IL-33 relative to MC were analyzed) with their associated receptors expressed on activated memory CD4^+^ T cells. the last column provides the gene upregulation (Log2 fold change) induced by IL-33 in MCs.
